# In-vivo transfection of pcDNA3.1-IGFBP7 inhibits melanoma growth in mice through apoptosis induction and VEGF downexpression

**DOI:** 10.1186/1756-9966-29-13

**Published:** 2010-02-16

**Authors:** Rong-Yi Chen, Hong-Xiang Chen, Jia-Xi Lin, Wei-Bing She, Ping Jiang, Li Xu, Ya-Ting Tu

**Affiliations:** 1 Department of Dermatology, Affiliated Union Hospital, Tongji Medical College, Huazhong University of Science and Technology, Wuhan, 430022 PR China; 2 Institute of Dermatology, Guangdong Medical College, Zhanjiang, 524023, PR China

## Abstract

**Background:**

Genome-wide RNA interference screening study revealed that loss of expression of insulin-like growth factor binding protein 7 (IGFBP7) is a critical step in development of a malignant melanoma (MM), and this secreted protein plays a central role in apoptosis of MM. In this study we constructed pcDNA3.1-IGFBP7 to obtain high expression of IGBPF7 and to inhibit the growth of MM in C57BL/6J mice.

**Methods:**

pcDNA3.1-IGFBP7 was transfected into B16-F10 cell, the expression of IGFBP7 was detected by RT-PCR and western blot. The proliferations and apoptosis rates of transfected and control cells were measured by CCK8 and FCM, respectively. The tumorigenicity and tumor growth in both pcDNA3.1-IGFBP7 group and control groups were studied in C57BL/6J mice model. IGFBP7, caspase-3, and VEGF expressions in tumor tissue were measured by immunohistochemistry. Apoptosis of tumors were detected by TUNEL.

**Results:**

We demonstrated this plasmid inhibited proliferation of B16-F10 melanoma cells efficiently *in vivo*, exploiting the high expression of IGFBP7. More importantly, in-vivo transfection of pcDNA3.1-IGFBP7 inhibited MM growth in C57BL/6J mice. The inhibition of MM growth was proved owing to apoptosis and reduced expression of VEGF induced by pcDNA3.1-IGFBP7.

**Conclusions:**

These results suggest a potential new clinical strategy for MM gene treatment.

## Background

MM is responsible for 80% of skin cancer deaths, and to date its incidence has been increasing. Although development of surgical, chemotherapeutic and radiotherapeutic treatment keeps ongoing, the 5-year survival rate of late stage MM patients is only 10-20% [1-4]. Therefore, a new effective therapy for MM is highly desired. In the previous studies, we demonstrated that the synthesis of vascular endothelial growth factor (VEGF) and growth of MM in xenograf models [3] were significantly inhibited by using small-interfering RNA (siRNA), which makes us believe that the modulation of aberrant signaling pathways in MM cell will probably provide more effective and potential nontoxic therapy for MM. However, this approach still has its shortcomings, in that *VEGF *is one of the downstream target genes of insulin-like growth factor (IGF), which is important in promoting tumor angiogenesis [5-8]. Although pU-VEGF-siRNA directly inhibited MM cell proliferation by reducing VEGF expression, it could not induce valid apoptosis.

Recently, immunohistochemical analysis of human skin, nevi, and melanoma samples implicates loss of IGFBP7 expression as a critical step in melanoma carcinogenicity [9]. Thus, the relationship between IGF axis and carcinogenesis has become one of the hottest spots. The IGF system is composed of IGFs, IGF receptors, and Insulin-like growth factor-binding proteins (IGFBPs). IGFBP7 belongs to the IGFBP superfamilies. It is also known as IGFBP-related protein 1 (IGFBP-rP1) or Mac25. It is a member of soluble protein family that binds IGFs with low affinity, and is expressed in a wide range of tissues [10,11]. In-vitro studies demonstrated that IGFBP7 induced the apoptosis of many cancer cells [12,13], e.g., breast and prostate cancer cells, and plays a potential tumor suppressor role against colorectal carcinogenesis. Moreover, Wajapeyee, [9] et al showed that recombinant IGFBP7 (rIGFBP7) induced apoptosis in melanoma cell lines, efficiently. These exciting data suggested that IGFBP7 may be an efficacious anticancer agent, since experiments have provided evidences that IGFBPs have both IGF-dependent and IGF-independent antitumoral actions [13,14].

Recent data also demonstrated that a prostatic carcinoma cell line stably transfected with IGFBP7 cDNA showed poor tumorigenicity both in vitro and in vivo [10]. Meanwhile, in our previous study, we found that IGFBP7 expression was low in B16-F10 cells. However, it is still unclear whether IGFBP7 cDNA inhibits proliferation of B16-F10 cells in vitro or B16-F10 MM growth in vivo. Therefore, in the present study, we constructed the pcDNA3.1-IGFBP7 plasmid as an antitumor agent to investigate whether it is effective in treating mice bearing B16-F10 melanoma tumor.

## Methods

### Plasmid construction

The pcDNA3.1-IGFBP7 expression plasmid was constructed. *IGFBP7 *gene (GenBank ID: 29817 No.AK156315.1) was amplified by RT-PCR from mRNA of splenocytes derived from C57BL/6J mice (IGFBP7 fw: 5'GAAGATCTATGGAGCGGCCGTCGCT-3', IGFBP7 rev: 5'-CGGAATTCTTTATAGCTCGGCACCTTCACCT-3'). *IGFBP7 *cDNA was purified by Shanghai Biological Engineering Company. The eukaryotic vector expressing eGFP and IGFBP7 was termed as pcDNA3.1-IGFBP7, and pcDNA3.1-CONTROL only expressed eGFP. The inserted sequences were verified by DNA sequencing, and digested by restriction endonuclease (EcoRI, and Bgl II enzyme).

### Tumor cells and in vitro transfection with pcDNA3.1-IGFBP7

B16-F10 cells were purchased from the Institute of Cell Biology (Shanghai institute for biological sciences). Cells were seeded in six-well plates (2 × 10^5 ^cells per well), cultured overnight at 37°C in 5% CO_2_, and grown to 60% confluence prior to transfection. Transfection with pcDNA3.1-IGFBP7 was performed by Effectene Transfection Reagent (QIAGEN Companies) according to the manufacturer's instructions. Cells transfected with pcDNA3.1-CONTROL and those without any transfection served as controls. The experimental and two control groups were termed pcDNA3.1-IGFBP7, pcDNA3.1-CONTROL and B16-F10 cells, respectively. All experiments were preformed in triplicate and repeated at least twice.

### RT-PCR and gelelectrophoresis

Total RNA from 1 × 10^6 ^cultured cells was extracted using the TRIZOL reagent (Invitrogen, San Diego, U.S.A.). Then RNA was reversely transcribed and synthesized to cDNA using Reverse transcriptase (Rever TraAce-α-TM, TOYOBO, JAPAN). Primer sequences for IGFBP7 (fw: 5'-GTAAGGAGGACGCTGGAGAGT-3', rev: 5'-CTGGCTGTAATAAAGTGTTAGTGG-3') and β-actin (fw: 5'-CCGTGAAAAGTGACCCAG-3' rev: 5'-TAGCCACGCTCGGTCAGG-3'). PCR and gelelectrophoresis conditions were described as previous [3]. The expected size of fragment of IGFBP7 and β-actin was 255 bp, 136 bp, respectively.

### Analysis of Cell Viability

Cell viability was determined by the Cell Counting Kit-8 (Dojindo Laboratories, Kumamoto, Japan) and measured by microplate reader scanning at 450 nm as previously described elsewhere [15].

### Quantification of cell apoptosis by flow cytometry

B16-F10 cells were washed by PBS and collected after digestion by 0.25% trypsin, cell suspension was added dropwise to PBS while gently vortexed, then centrifuged at 1000 rpm at 4°C for 10 min. After resuspension of the cells in labeling buffer, 10 μl Annexin VFITC was added and then incubated in the dark. Following 150 μL of propidium iodide (PI) was added, the cells were incubated for 2 h at room temperature. Then cell apoptosis was measured by flow cytometry [16,17].

### Mice

Thirty-six six-week-old female Wild-type C57BL/6J mice weighing 18-25 g were treated in accordance with the guidelines of the National Institutes of Health for the humane treatment of animals, and all animal protocols were approved by Huazhong University of Science and Technology's animal care and use committee. Mice were anesthetized with urethane (1.9 g/kg sc; 12.5 mg urethane/ml 0.9% saline; Sigma Chemical, St. Louis, MO), and their temperature was maintained at 37°C[18]. 1 × 10^4 ^B16-F10 cells were injected subcutaneously in the lower backs of mice, where MM emerged after 1 week. Tumor volume (v) was calculated as follow, v = L × I^2 ^× 0.5^2^, where L and I represent the maximum and minimum tumor diameter measured weekly. All the mice were divided into three groups randomly (n = 12 each group), termed pcDNA3.1-IGFBP7, pcDNA3.1-CONTROL and B16-F10 cells groups respectively. Then Invivofectamine reagent-plasmid duplex complexes 200 μl (Reagent for in vivo plasmid delivery, Invitrogen, U.S.A), containing pcDNA3.1-IGFBP7 (1 μg), or pcDNA3.1-CONTROL (1 μg), DMEM 200 μl were respectively injected into the tumors for every 3 day. The delivery efficiency was evaluated by GFP fluorescence and RT-PCR. After 3 weeks the mice were killed (with permission of the Animal Protection Association of Tongji Medical College). Tumors were cryosectioned or fixed in 10% buffered formalin and embedded in paraffin detected by immunohistochemistry.

### Western blot analysis

IGFBP7 expression changes within mouse xenografts were checked by western blotting as described previously [19], the antibodies to IGFBP7 and β-actin were purchased from (R&D systems U.S.A.).

### Detection of IGFBP7, caspase-3, VEGF by immunohistochemistry or laser scanning confocal microscopy

Detection is based on the formation of the Avidin-Biotin Complex (ABC) with primary antibodies that reacted with tissue antigens. Primary antibodies were listed as follows: IGFBP7(1:25 R&D systems U.S.A MAB21201), caspase-3(1:20 R&D systems U.S.A MAB835), VEGF (1:20 Santa Cruz Biotechnology, sc-7269). Coverslips containing pcDNA3.1-IGFBP7, pcDNA3.1-CONTROL tumor section were mounted onto glass slides and observed with a Zeiss 510 confocal microscope. Green fluorescent protein and TRITC-labeled IGFBP7 were viewed through the GFP, and tetramethyl rhodamine isothiocyanate (TRITC) fluorescence channel, respectively. Appropriate positive and negative controls were included. The expression of caspase-3 and VEGF visualization is based on enzymatic conversion of a chromogenic substrate (AEC), (CTS018 R&D systems U.S.A). No significant difference in intensity of immunohistochemical staining was designated as negative (0), positive (1), strong positive (2) and the percentage of positive cells was scored as less than 5% (0), 5%~25% (1), 26%~50% (2), 51%~75% (3) or over 75% (4) of cells stained[20]. Values in the parentheses were multiplied together to the scores for IGFBP7, caspase-3, VEGF expression.

### Detection of tumor apoptosis

Tumor apoptosis was detected using terminal deoxynucleotidyl transferase-mediated deoxyuridine triphosphate nick end labelling (TUNEL, Catalog # 11684809910, ROCHE Germany) according to the supplier's instructions, and apoptosis index (AI) was used to evaluate cell apoptosis.

### Statistics

The statistical analysis was performed using SPSS 13.0 software (SPSS, Chicago, IL, U.S.A.). Statistical comparisons of mean values were performed using Student's t-test and Kruskal-Wallis Test, the correlations was analyzed by Spearman's rho correlation analysis. All P-values were determined from two-sided tests. A significance criterion of *P *< 0.05 was used in these studies.

## Results

### Identification of pcDNA3.1-IGFBP7 plasmid

The sequence analysis of constructed pcDNA3.1-IGFBP7 by a DNA sequencer showed the same sequence of eukaryotic IGFBP7 mRNA as designed. Meanwhile, recombinant pcDNA3.1-IGFBP7 plasmid was confirmed by restriction enzyme analysis, as shown in additional files 1, Figure S1. These results indicated that the pcDNA3.1-IGFBP7 vector was constructed successfully. Then pcDNA3.1-IGFBP7 and pcDNA3.1-CONTROL were transfected into cells successfully, termed pcDNA3.1-IGFBP7 cells and pcDNA3.1-CONTROL cells, respectively with transfection rate being about 60%, as shown in additional files 1, Figure S2.

### Effect of pcDNA3.1-IGFBP7 plasmid on IGFBP7 expression

It was found that the *IGFBP7 *mRNA levels in pcDNA3.1-IGFBP7-transfected B16-F10 cells were increased by about 4-fold, 8-fold, 7-fold, 6-fold on days 1, 3, 6 and 12, respectively, compared with the control group. But no change of IGFBP7 expression in pcDNA3.1-CONTROL groups (*P *> 0.05) was found, suggesting that pcDNA3.1-IGFBP7 vector specifically promotes expression of IGFBP7 without effects on *β-actin *mRNA,, as shown in additional files 2, Figure S1. Meanwhile, the expression of IGFBP7 was detected by western blot. The western blot showed that pcDNA3.1-IGFBP7 increased the expression of IGFBP7. Results are consistent with previous determined by RT-PCR. According to these results detected by RT-PCR and western blot, the IGFBP7 expressed in the pcDNA3.1-IGFBP7 group were significantly higher in the pcDNA3.1-CONTROL and B16-F10 cells groups (*p *< 0.03), as shown in additional files 2, Figure S2.

### pcDNA3.1-IGFBP7 suppresses B16-F10 cells growth in vitro

The proliferation of pcDNA3.1-IGFBP7-transfected cells was significantly suppressed compared with control cells (*P *< 0.01). The highest suppression effect of pcDNA3.1-IGFBP7 was found at 48 h post-transfection, and no significant difference in proliferation between pcDNA3.1-CONTROL and untransfected cells was observed (*P *> 0.05), indicating that transfection of pcDNA3.1-IGFBP7 blocks the proliferation of B16-F10 cells by increasing IGFBP7 synthesis and secretion, as shown in additional files 2, Figure S3. To evaluate apoptosis-induced effect of pcDNA3.1-IGFBP7 in melanoma cells, B16-F10 cells at 48 h post-transfection was monitored by FCM. The apoptosis rate in pcDNA3.1-IGFBP7 group (24.6%) was significantly higher than that in control groups (*P *< 0.01). However, no marked apoptosis was observed in pcDNA3.1-CONTROL (6.1%) and B16-F10 groups (5.3%). Our finding mentioned above indicates that the long-term IGFBP7 expression possibly establishes a desirable basis for the therapeutic effect *in vitro*.

### Effect of pcDNA3.1-IGFBP7 on IGFBP7 expression and growth of MM homeograft in vivo

To evaluate the therapeutic potential of pcDNA3.1-IGFBP7 on B16-F10 MM homeograft *in vivo*, we performed intratumoral injection of pcDNA3.1-IGFBP7 to study the effect on carcinogenesis. The results showed that pcDNA3.1-IGFBP7 inhibited tumor growth, at the time of killing, the volumes of MM in B16-F10 cell group and pcDNA3.1-CONTROL group were 587 ± 35 mm^3 ^and 566 ± 34 mm^3^, respectively, being about 6-fold increase over the starting volume; whereas the volume of B16-F10 tumors injected with pcDNA3.1-IGFBP7 were 256 ± 25 mm^3^, with the volume increase being only 2.8-fold. The delay in tumor growth was statistically significant (*P *< 0.001). To evaluate the expression of IGFBP7 in tumor homeograft, the proteins were determined by western blotting. IGFBP7 expression in the pcDNA3.1-IGFBP7 group was significantly higher than in pcDNA3.1-CONTROL and B16-F10 cells groups (*p *< 0.01), whereas there was no significant difference in IGFBP7, expression was found between pcDNA3.1-CONTROL and B16-F10 cells groups (*p *> 0.05). Transfection of pcDNA3.1-IGFBP7 *in vivo *not only inhibited MM growth in C57BL/6J mice, but also prolonged C57BL/6J mice survival bearing B16-F10 melanoma tumor.

### Effect of pcDNA3.1-IGFBP7 on IGFBP7, caspase-3, VEGF and apoptosis expression in vivo

**Figure 1:**
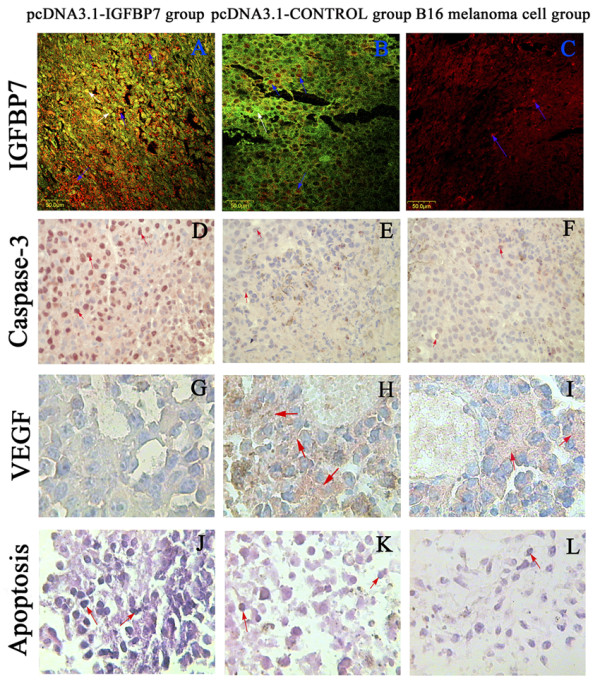
**Detection of IGFBP7, caspase-3, VEGF, and apoptosis expressed in homeograft tumors sections with original magnification × 100 in A-F, and ×400 in G-L**. A shows significantly higher IGFBP7 expression in pcDNA3.1-IGFBP7. B demonstrates the successful transfection of pcDNA3.1 plasmid. C shows the physiological expression of IGFBP7 in melanoma (red color, as blue arrows indicate). D-F shows the effect of pcDNA3.1-IGFBP7 on caspase-3 expression in the cytoplasm of tumor sections, with strong expression in pcDNA3.1-IGFBP7 group seen in D, while weak expression in the pcDNA3.1-CONTROL and B16-F10 cell groups seen in E, F. G-I shows the expression of VEGF *in vivo*, with negative expression in most of cells in the pcDNA3.1-IGFBP7 group seen in G, while strong expression in the cytoplasm of pcDNA3.1-CONTROL and B16-F10 cell groups (red arrow represented) showed in H, I. J-L shows tumor apoptosis *in vivo*, with few apoptotic cells in pcDNA3.1-CONTROL (K) and B16-F10 cell groups (L), while increased apoptosis in the B16-F10 tissue in mice treated with pcDNA3.1-IGFBP7 (J) (red arrow shows deep blue cells).

To investigate the effect of pcDNA3.1-IGFBP7 on IGFBP7, caspase-3, VEGF expression, and MM apoptosis *in vivo*, we performed fluorescent immunohistochemistry and cytometry. As shown in Fig. 1. IGFBP7 and caspase-3, VEGF were mainly expressed in the cytoplasm of tumor cells. IGFBP7 was determined by fluorescent immunohistochemistry, positive staining of TRITC labeled IGFBP7 protein is red and localized in the cytoplasm, while GFP protein expressed by plasmids is green. The expression of caspase-3 and VEGF visualization is based on AEC staining. The results are consistent with our hypothesis, as show in Fig. 1. A-F that IGFBP7 and caspase-3 expression in the pcDNA3.1-IGFBP7 group is significantly higher in the pcDNA3.1-CONTROL and B16-F10 cells groups (IGFBP7 *P *< 0.002, caspase-3 *p *< 0.004), but VEGF expression in the pcDNA3.1-IGFBP7 group is significantly lower in the pcDNA3.1-CONTROL and B16-F10 cells groups (*P *< 0.006) (Fig. 1. G-I) respectively, and no significant difference in IGFBP7 and caspase-3. VEGF expression is found between the pcDNA3.1-CONTROL and B16-F10 cells groups (*P *> 0.05). According to these results determined by immunohistochemistry, there were significantly more apoptotic cells in the pcDNA3.1-IGFBP7 group than in the pcDNA3.1-CONTROL and B16-F10 cells groups (*p *< 0.031). As shown in Fig. 1. J-L, morphological characters of apoptotic cells are cell shrinkage, deformation, and loss of contact with neighbouring cells. Fig. 1. J shows more apoptotic cells in the pcDNA3.1-IGFBP7 group than in the pcDNA3.1-CONTROL (Fig. 1. K), and B16-F10 cells groups (Fig. 1. L), which contained almost the same numbers of apoptotic cells. The expression of IGFBP7 is positively correlated with caspase-3, and cell apoptosis rate (r_s _= 0.704, r_s _= 0.806 respectively, *p *< 0.01). However there is negative correlation between IGFBP7 and VEGF r_s _= -0.564, *p *< 0.01). These results suggested that pcDNA3.1-IGFBP7 inhibited the proliferation of MM cells by up-regulating IGFBP7 and caspase-3 expression and down-regulating VEGF expression *in vivo*, resulting in slowing down of MM growth.

As to show the exactitude of our experiment design, we used pcDNA3.1-IGFBP7 simultaneously expressed GFP and IGFBP7 rather than pcDNA3.1 plasmid containing only *IGFBP7 *gene. That was because, if we used pcDNA3.1 plasmid only containing *IGFBP7 *gene, we could not estimate the transfection efficiency *in-vivo *experiments, and moreover, we could not discriminate whether high level of IGFBP7 expression in xenograft sections dued to plasmid transfection or physiological IGFBP7 synthesis of melanoma. Well, pcDNA3.1-IGFBP7 simultaneously expressed GFP and IGFBP7 could solve both of the problems, as shown in additional files 3, Figure S2. We evaluated apoptosis-induced effect in melanoma cells of pcDNA3.1 only containing IGFBP7 gene, and in those of pcDNA3.1-IGFBP7 simultaneously expressed GFP and IGFBP7, finding out that insersion of GFP would not affect the expression of IGFBP7, as shown in additional files 3, Figure S1.

## Discussion

It has been confirmed that transfection with anti-tumor plasmids is more specific, more efficient, and longer lasting for anti-tumor therapy than recombinant protein. Transfection of anti-tumor plasmids may have some advantages over the application of rIGFBP7, namely the less danger of immunological rejection and the low cost of synthesis and purification [3]. In addition, MM cells transfected with eukaryotic expression plasmids could have stable and effective expression of IGFBP7 gene. Our research demonstrated that pcDNA3.1-IGFBP7 vector promotes expression of IGFBP7 specifically and have a long-lasting effect. However, it is conflicting to our hypothesis that IGFBP7 expression should ascensus, but it was attenuate over time. The possible explanation for this phenomenon was attributed to the high performance of P_CMV _promoter contained in pcDNA3.1-IGFBP7, which would exhaust and be toxic to tumor cells since it ad infinitum synthesized IGFBP7. Meanwhile augmentation of IGFBP7 in cell supernatant would induce apoptosis of part of tumor cells and therefore, the synthesis of IGFBP7 also decreases with reduction of tumor cells.

To determine therapeutic potential of pcDNA3.1-IGFBP7 *in vitro*, we analyzed cells viability and apoptosis rates by the Cell Counting Kit-8 and FCM. Our results are consistent with the research of Sprenger [13], which indicated that the growth of a tumorigenic SV40 prostate cell line, M12, was suppressed by transfecting the IGFBP-rP1 cDNA. Also, prostatic carcinoma cells were stably transfected with IGFBP7 cDNA and showed poor tumorigenicity [21]. Moreover, IGFBP7 which acts through autocrine/paracrine pathways to inhibit BRAF-MEK-ERK signaling and induce apoptosis [9], but it is contradictory to some researcher's findings, as they indicated that IGFBP7 was highly overexpressed in glioma tissues, mediateing glioma cell growth, and migration [22]. In addition, the expression pattern of IGFBP7 varies with tumor types. Both up-regulated expression [23] and down-regulated expression [24,25] of IGFBP7 is observed in different types of cancer. In our previous study, we found that IGFBP7 expression was low in B16-F10 cells. Vladislava [26] also indicated that unlike human melanomas, the murine melanoma cell lines (B16-F10) did not have activating mutations in the Braf oncogene at exon 11 or 15, however, there were distinct patterns of mutation in the ras gene. RAS proteins are membrane-bounded small G proteins, and RAF, MEK, and ERK are cytosolic protein kinases that form a tiered protein kinase cascade downstream of RAS, whereas ARAF and CRAF are not mutated because their regulation is fundamentally different from that of BRAF. As a consequence, RAS is mutated in melanoma, the cells (B16-F10) switch their signaling from BRAF to CRAF [27], then IGFBP7 expression is decreased, enabling the cells to escape from senescence and resulting in uncontrolled proliferation. Accordingly, RAS-CRAF-MEK-ERK pathways contribute to the development of murine melanoma. Transfection of pcDNA3.1-IGFBP7 into B16-F10 cells, upgraded the expression of IGFBP7, which inhibits CRAF-MEK-ERK signaling through an autocrine/paracrine pathway, thereby restraining proliferation and activates apoptosis. Together, these results suggest that IGFBP7 plays different roles in different tumor or host environments. Therefore, we need to evaluate the therapeutic potential of pcDNA3.1-IGFBP7 on B16-F10 *in vivo*.

Although the apoptosis-inducing effect of pcDNA3.1-IGFBP7 in cultured cells was shown for *in vitro *applications, its therapeutic applications *in vivo *represent an altogether more daunting challenge. To elevate transfection efficiency, we employed Invivofectamine (a new in vivo plasmid delivery reagent) to carry pcDNA3.1-IGFBP7 transfected into tumors tissue. Fortunately, our data clearly showed that intratumoral injection of the Invivofectamine pcDNA3.1-IGFBP7 complex was able to slow down the growth of B16-F10 MM homograft, and its transfection efficiency was about 70%. Most importantly, it had a lasting effect on tumor development, being effective for at least 20 days, because stable expression of IGFBP7 by using pcDNA3.1-IGFBP7. We focused on the therapeutic mechanisms of the Invivofectamine pcDNA3.1-IGFBP7 complex in B16-F10 MM homograft. The antitumor research of IGFBP has provided evidence that IGFBPs may have both IGF-dependent and independent actions. We hypothesized that IGFBP7 can inhibit MM gowth by IGF-dependent way [14], and reduce VEGF expression through preventing IGF-Ibinding to its receptors. In addition, IGFBP7 induces MM apoptosis through a novel IGF-independent pathway. To confirm the presumption, we studied IGFBP7, caspase-3, VEGF expression and apoptosis in tumor homograft tissues. The results of the immunohistochemistry and TUNEL showed that, IGFBP7 and caspase-3 expression in pcDNA3.1-IGFBP7 group are significantly higher than in pcDNA3.1-CONTROL and B16-F10 cells groups, but VEGF expression in the pcDNA3.1-IGFBP7 group were significantly lower than both in control groups, and no significant difference in IGFBP7 and caspase-3, VEGF expression were found between the pcDNA3.1-CONTROL and B16-F10 cells groups. According those results determined by immunohistochemistry, there were significantly more apoptotic cells in the pcDNA3.1-IGFBP7 group than in control groups. This was considered possibly to relate to IGFBP7 promote apoptosis effectiveness. However, our finding contrasted with the results of Adachi [28] et al, who found that high expression of IGFBP7 in invasive tumor cells was associated with poor prognosis. This discrepancy may be due to the difference in the immunohistochemical scoring [20,29]. We used the composite score to evaluate the expression of IGFBP7, which seems to be one of the most promising and accurate scoring systems currently defined.

Futhermore, we demonstrated that the expression of IGFBP7 is positive correlation with caspase-3, and cell apoptosis rate. In addition, there is negative correlation between IGFBP7 and VEGF. Those results suggested that pcDNA3.1-IGFBP7 can up-regulate IGFBP7, caspase-3 expression, and down-regulate VEGF expression *in vivo *to inhibit the proliferation of MM cells, which resulted in slowing down of MM growth, as shown in additional files 4. Angiogenesis is essential for tumor development, and the increasing evidences show that IGF-I plays a crucial role in tumor growth by up-regulating the VEGF expression and neovascularisation [30]. A recent study indicated that IGFBP7 might exhibit angiogenesis-modulating properties, reducing VEGF expression by regulating IGF availability in body fluids and tumor tissues and modulating combination of IGF-I to its receptors [30,31]. Moreover the reduction of VEGF-induced tube formation by IGFBP7 could be mainly mediated by inhibition of MAP kinase cascade through c-Raf, and BRAF-MEK-ERK signalling [32], Although our research implied IGFBP7 blocks VEGF-induced angiogenesis and VEGF expression by interfering with IGF-I, its role in tumor angiogenesis remains poorly understood. The mechanisms by which IGFBP7 induced apoptosis and inhibit neovascularization should be further explored.

## Conclusion

Our data show that increasing IGFBP7 expression by using the pcDNA3.1-IGFBP7 plasmid suppresses MM growth, induces apoptosis and reduces VEGF *in vitro *and *in vivo*. Intratumoral injection of pcDNA3.1-IGFBP7 holds promise as a clinical gene therapy approach for MM, which provide a framework for further studies of its broader applicability to a range of human tumors. However, there are several insufficiencies on this therapeutics. Firstly, it would be difficult to make uniform distribution of pcDNA3.1-IGFBP7 in tumor tissue by intratumoral injection of invivofectamin, and a transferrin-polyethylenimine (Tf-PEI) delivery system (our previous studies) needs to be used in the further study. Secondly, there are no suitable MM cell lines available that express high level of IGFBP7 to prove the specificity of anti-tumor effect of pcDNA3.1-IGFBP7. Moreover, many biological roles of pcDNA3.1-IGFBP7 remain to be elucidated.

## Abbreviations

IGFBP7: insulin-like growth factor binding protein 7; MM: malignant melanoma; VEGF: vascular endothelial growth factor; TUNEL: terminal deoxynucleotidyl transferase-mediated deoxyuridinetriphosphate nick end labelling.

## Competing interests

The authors declare that they have no competing interests.

## Authors' contributions

RC carried out the design of the study and molecular biological experiments; HC drafted the manuscript; JL performed the statistical analysis; PJ carried out the pathologic examination studies and western blot analysis; WS carried out the animal experiments; LX carried out the RT-PCR and immunohistochemistry; YT carried out the design of the study. All authors read and approved the final manuscript.

## Supplementary Material

Additional file 1 **pcDNA3.1-IGFBP7 plasmid checked by restriction enzyme analysis, and transfection with Effectene authenticated by immunofluorescence**. Restriction enzyme analysis of pcDNA3.1-IGFBP7 plasmid by EcoR I and Bgl II manifested that the obtained plasmid was the objective one with predicted length. Plasmid transfection with Effectene was successful, authenticated by immunofluorescence. Click here for file

Additional file 2 **Effect of pcDNA3.1-IGFBP7 plasmid on IGFBP7 expression in vitro**. Higher concentration of pcDNA3.1-IGFBP7 plasmid led to higher IGFBP7 mRNA and protein expression in B16-F10 melanoma cells, detected by RT-PCR and western blot. pcDNA3.1-IGFBP7 transfection led to reduction of B16-F10 cells viability, determined by the Cell Counting Kit-8. Click here for file

Additional file 3 **Effect of different plasmids on tumor cell apoptosis rate detected by flow cytometry and laser scanning confocal microscopy**. Apoptosis rate detected by flow cytometry of B16 melanoma resulted in an obvious increase in pcDNA3.1-IGFBP7 group than those in pcDNA3.1-CONTROL and B16 groups, consistent with laser confocal display of tumor sections of the three groups, suggested significant effects of in-vitro and in-vivo pcDNA3.1-IGFBP7 transfection on B16 apoptosis. Click here for file

Additional file 4 **In-vivo anti-tumor effect of pcDNA3.1-IGFBP7 plasmid**. Survival curves and tumor volumes showed different effects of the three groups. pcDNA3.1-IGFBP7 group has a significantly higher survival rate and smaller tumor size, compared to pcDNA3.1-CONTROL and B16-F10 groups. Click here for file
