# Supplementary material for: In-vivo transfection of pcDNA3.1-IGFBP7 inhibits melanoma growth in mice through apoptosis induction and VEGF downexpression
Source: J Exp Clin Cancer Res. 2010 Feb 16;29(1):13. doi: 10.1186/1756-9966-29-13 (PMC2844372; doi:10.1186/1756-9966-29-13)
Supplement: Additional file 1 — pcDNA3.1-IGFBP7 plasmid checked by restriction enzyme analysis, and transfection with Effectene authenticated by immunofluorescence. Restriction enzyme analysis of pcDNA3.1-IGFBP7 plasmid by EcoR I and Bgl II manifested that the obtained plasmid was the objective one with predicted length. Plasmid transfection with Effectene was successful, authenticated by immunofluorescence. [file 1756-9966-29-13-S1.PDF]

Additional file 1

pcDNA3.1-IGFBP7 plasmid checked by restriction enzyme analysis, and transfection with Effectene authenticated by immunofluorescence.

Restriction enzyme analysis of pcDNA3.1-IGFBP7 plasmid by EcoR I and BglⅡ manifested that the obtained plasmid was the objective one with predicted length. Plasmid transfection with Effectene was successful, authenticated by immunofluorescence.

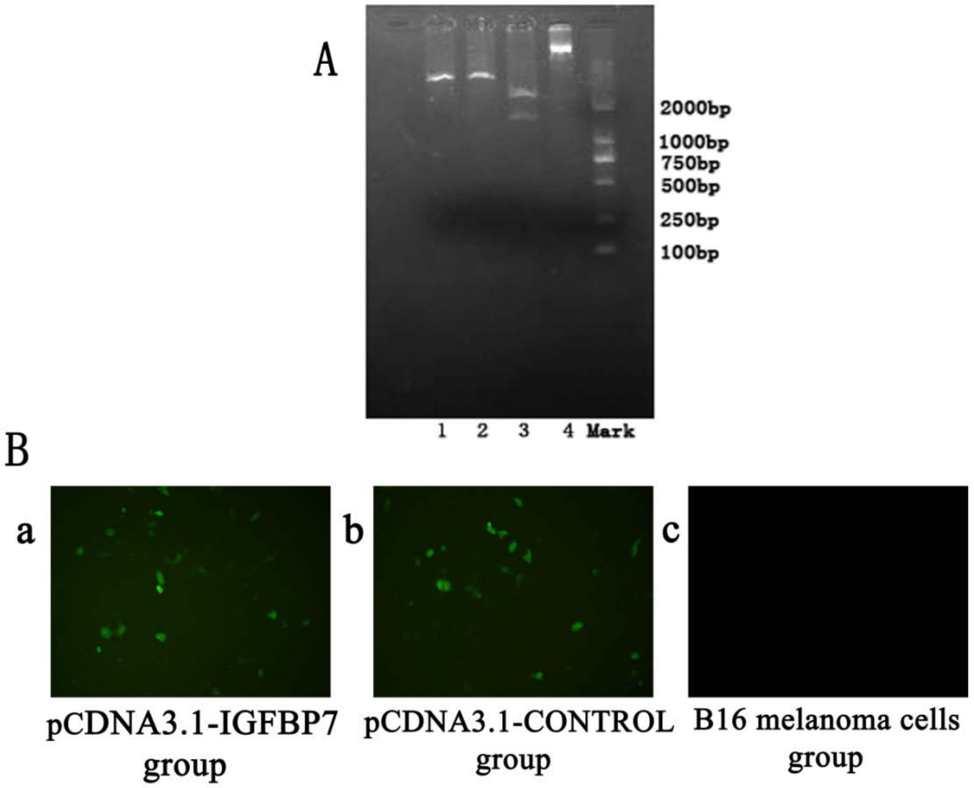

Figure S1 (A) pcDNA3.1-IGFBP7 plasmid was checked by restriction enzyme analysis M: Marker; 1: pcDNA3.1-IGFBP7 digested by BglⅡ; pcDNA-IGFBP7 digested by EcoR I ; 3: pcDNA3.1-IGFBP7 digested by EcoR I and BglⅡ; 4: pcDNA3.1 -IGFBP7, and **S2 (B)** pcDNA3.1-IGFBP7 and pcDNA3.1-CONTROL were transfected by Effectene successfully.
