# Supplementary material for: In-vivo transfection of pcDNA3.1-IGFBP7 inhibits melanoma growth in mice through apoptosis induction and VEGF downexpression
Source: J Exp Clin Cancer Res. 2010 Feb 16;29(1):13. doi: 10.1186/1756-9966-29-13 (PMC2844372; doi:10.1186/1756-9966-29-13)
Supplement: Additional file 2 — Effect of pcDNA3.1-IGFBP7 plasmid on IGFBP7 expression in vitro. Higher concentration of pcDNA3.1-IGFBP7 plasmid led to higher IGFBP7 mRNA and protein expression in B16-F10 melanoma cells, detected by RT-PCR and western blot. pcDNA3.1-IGFBP7 transfection led to reduction of B16-F10 cells viability, determined by the Cell Counting Kit-8. [file 1756-9966-29-13-S2.PDF]

Additional file 2

Effect of pcDNA3.1-IGFBP7 plasmid on IGFBP7 expression in vitro

Higher concentration of pcDNA3.1-IGFBP7 plasmid led to higher IGFBP7 mRNA and protein expression in B16-F10 melanoma cells, detected by RT-PCR and western blot. pcDNA3.1-IGFBP7 transfection led to reduction of B16-F10 cells viability, determined by the Cell Counting Kit-8.

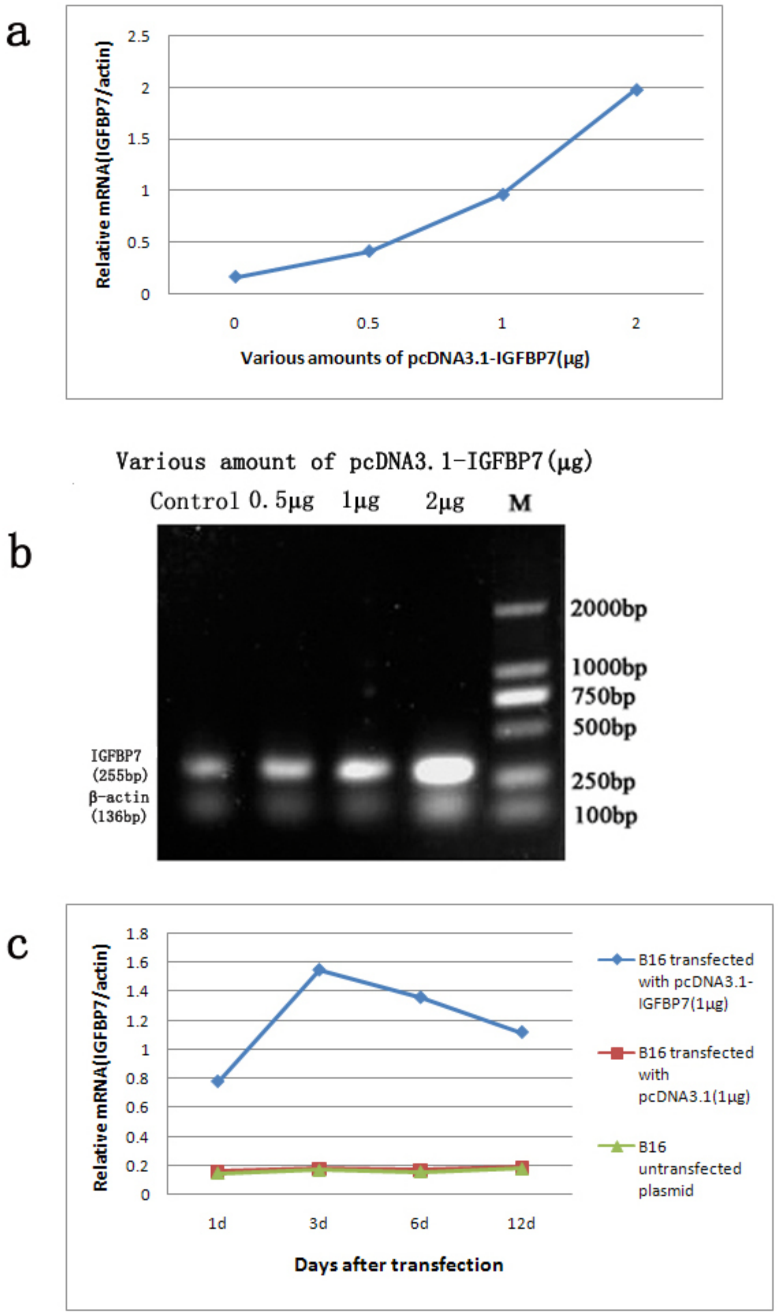

Figure S1 Effect of pcDNA3.1-IGFBP7 plasmid on IGFBP7 mRNA expression in B16-F10 melanoma cells

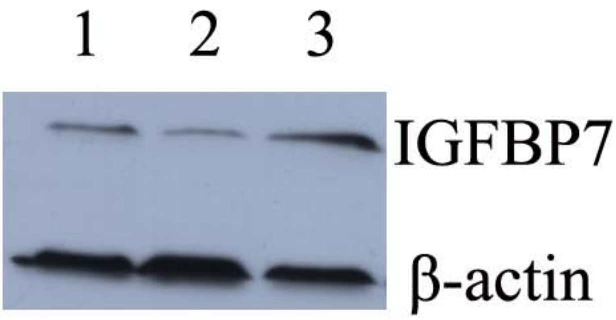

Figure S2 1 represents basic IGFBP7 expression in B16-F10 cell group 2 represents IGFBP7 expression of pcDNA3.1-CONTROL group 3 represents IGFBP7 expression of pcDNA3.1- IGFBP7 group

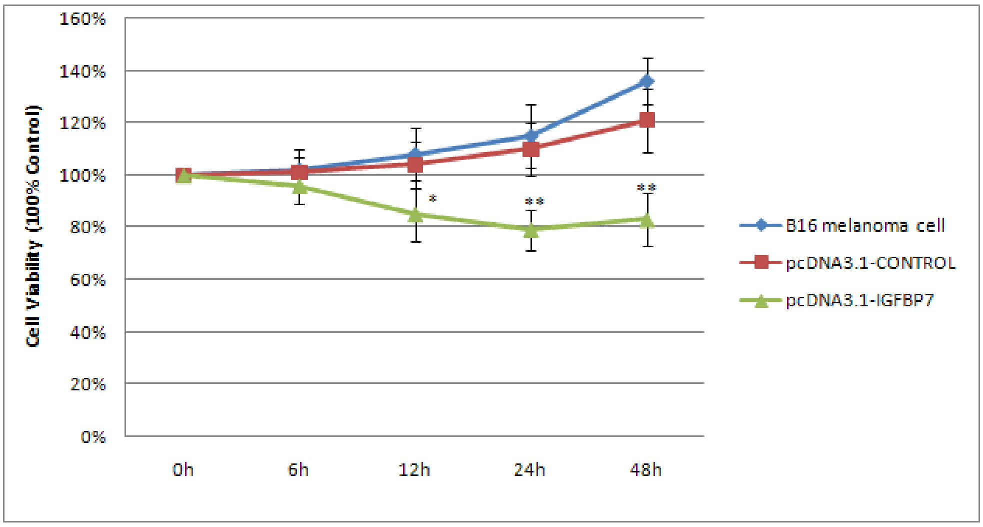

Figure S3 Cell viability of B16-F10 melanoma cell untransfected or transfected with pcDNA3.1-IGFBP7 (1μg), pcDNA3.1-CONTROL (1μg)
