# Supplementary material for: In-vivo transfection of pcDNA3.1-IGFBP7 inhibits melanoma growth in mice through apoptosis induction and VEGF downexpression
Source: J Exp Clin Cancer Res. 2010 Feb 16;29(1):13. doi: 10.1186/1756-9966-29-13 (PMC2844372; doi:10.1186/1756-9966-29-13)
Supplement: Additional file 3 — Effect of different plasmids on tumor cell apoptosis rate detected by flow cytometry and laser scanning confocal microscopy. Apoptosis rate detected by flow cytometry of B16 melanoma resulted in an obvious increase in pcDNA3.1-IGFBP7 group than those in pcDNA3.1-CONTROL and B16 groups, consistent with laser confocal display of tumor sections of the three groups, suggested significant effects of in-vitro and in-vivo pcDNA3.1-IGFBP7 transfection on B16 apoptosis. [file 1756-9966-29-13-S3.PDF]

Additional file 3

Effect of different plasmids on tumor cell apoptosis rate detected by flow cytometry and laser scanning confocal microscopy

Apoptosis rate dectectd by flow cytometry of B16 melanoma resulted in an obvious increase in pcDNA3.1-IGFBP7 group than those in pcDNA3.1-CONTROL and B16 groups, consistent with laser confocal display of tumor sections of the three groups, suggested significant effects of in-vitro and in-vivo pcDNA3.1-IGFBP7 transfection on B16 apoptosis.

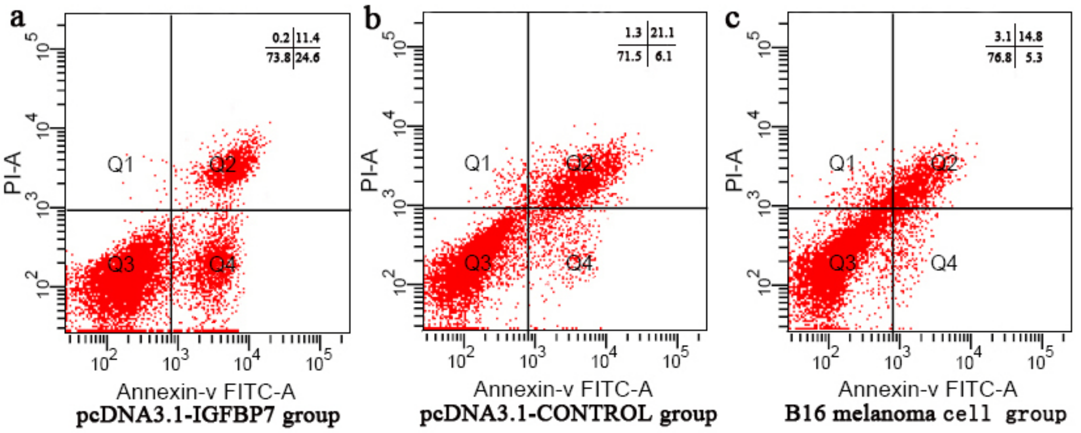

Figure S1 Apoptosis rate of B16 melanoma at 24h post-transfection (a).pcDNA3.1-IGFBP7 cell, 24.6%, (b) pcDNA3.1-CONTROL cell, 6.1%, (c) B16 melanoma cell, 5.3%.

We also use pcDNA3.1 plasmid only containing IGFBP7 gene *in vitro* transfection experiment, as shown in **Figure S2.** But if we used pcDNA3.1 plasmid only containing IGFBP7 gene, we could not estimate the transfection efficiency *in vivo* experiments. Moreover we can't discriminate whether high level of IGFBP7 expression in xenograft sections was due to plasmid transfection or melanoma

synthesis IGFBP7 physiologically. To estimate the transfection efficiency after intratumoral injection with pcDNA3.1-IGFBP7 *in vivo* and to demonstrate that xenograft plasmid transfection expressed high level of IGFBP7, we used pcDNA3.1-IGFBP7（simultaneously expressed GFP and IGFBP7）, as shown in **Figure S3**

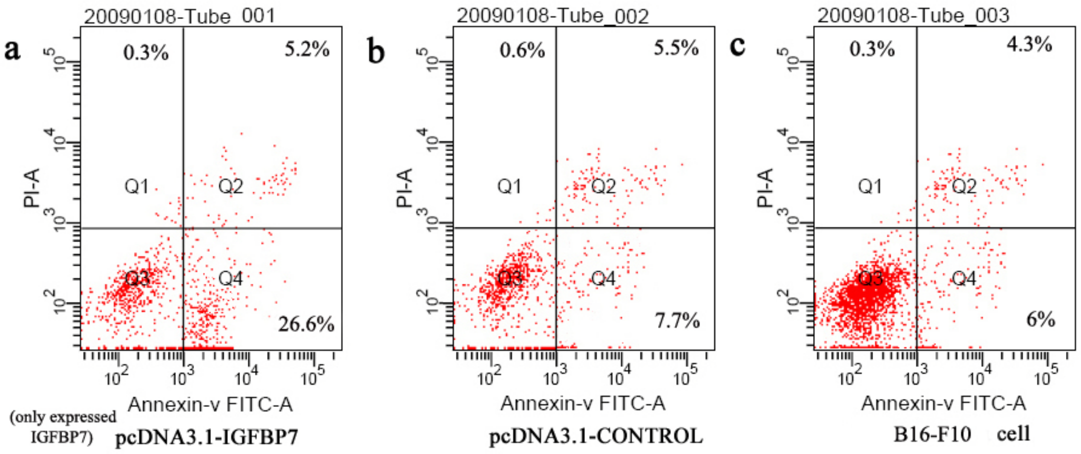

Figure S2 To evaluate apoptosis-induced effect in melanoma cells of pcDNA3.1 only containing IGFBP7 gene, B16-F10 cells at 48h post-transfection was monitored by FCM. The results were consistent with pcDNA3.1-IGFBP7 (simultaneously expressed GFP and IGFBP7).

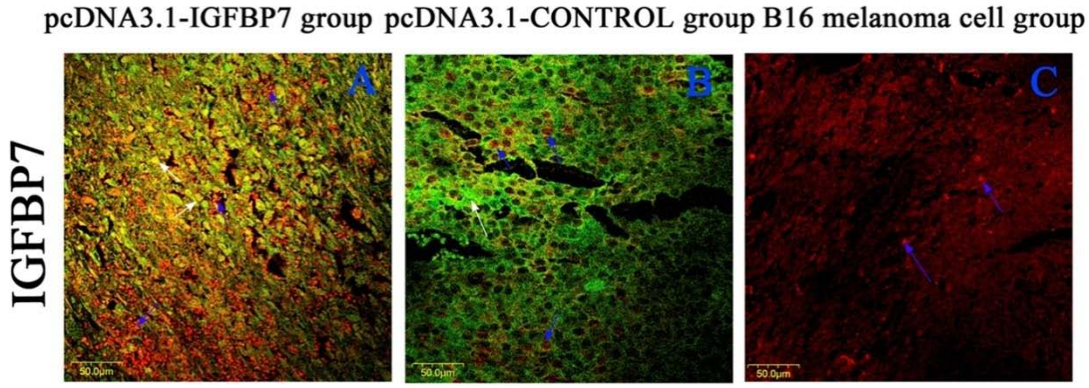

Figure S3 TRITC labelled IGFBP7 protein is red and localized in the cytoplasm, and GFP protein expressed by plasmid is green. We can see form A, the higher GFP fluorescence intensity, the higher IGFBP7 expressed in pcDNA3.1-IGFBP7 group

（yellow color area represents co-expressed GFP and IGFBP7）. It demonstrated high level of IGFBP7 expressed in xenograft sections were due to transfected with plasmid (simultaneously expressed GFP and IGFBP7). Although （figure B） pcDNA3.1-CONTROL group green fluorescence intensity was similar to figure A，the transfection efficiency were about 60~70%, but IGFBP7 expression (red color) was low. It means expression of IGFBP7 in the pcDNA3.1-IGFBP7 group is significantly higher than in the pcDNA3.1-CONTROL and B16-F10 cell groups (there isn't green fluorescence in figure C, owing to without transfected plasmid). From the results above, we can draw a conclusion that intratumoral injection of pcDNA3.1-IGFBP7 could increase IGFBP7 expression specifically, pcDNA3.1-CONTROL can't synthesis IGFBP7.
