# Supplementary material for: In-vivo transfection of pcDNA3.1-IGFBP7 inhibits melanoma growth in mice through apoptosis induction and VEGF downexpression
Source: J Exp Clin Cancer Res. 2010 Feb 16;29(1):13. doi: 10.1186/1756-9966-29-13 (PMC2844372; doi:10.1186/1756-9966-29-13)
Supplement: Additional file 4 — In-vivo anti-tumor effect of pcDNA3.1-IGFBP7 plasmid. Survival curves and tumor volumes showed different effects of the three groups. pcDNA3.1-IGFBP7 group has a significantly higher survival rate and smaller tumor size, compared to pcDNA3.1-CONTROL and B16-F10 groups. [file 1756-9966-29-13-S4.PDF]

Additional file 4

In-vivo anti-tumor effect of pcDNA3.1-IGFBP7 plasmid

Survival curves and tumor volumes showed different effects of the three groups. pcDNA3.1-IGFBP7 group has a significantly higher survival rate and smaller tumor size, compared to pcDNA3.1-CONTROL and B16-F10 groups.

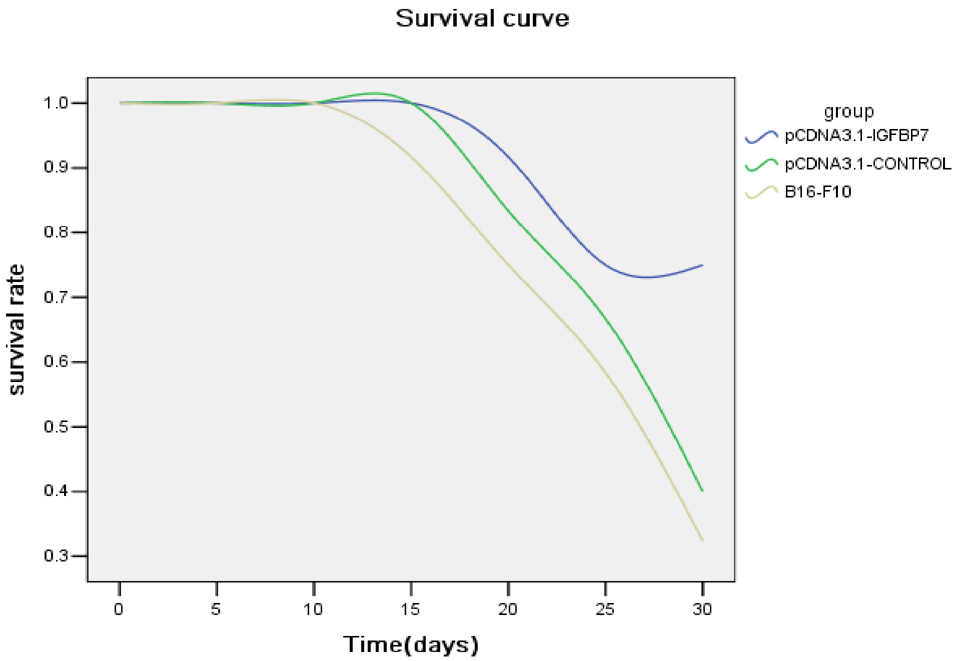

Figure S1 The C57/6J mice survival rate of pcDNA3.1-IGFBP7, pcDNA3.1-CONTROL, and B16-F10 groups at 25 days, were 75%, 50%, 41.7%, respectively. The result implies transfection of pcDNA3.1-IGFBP7 in vivo can prolong C57BL/6J mice survival, which bearing B16-F10 melanoma tumor.

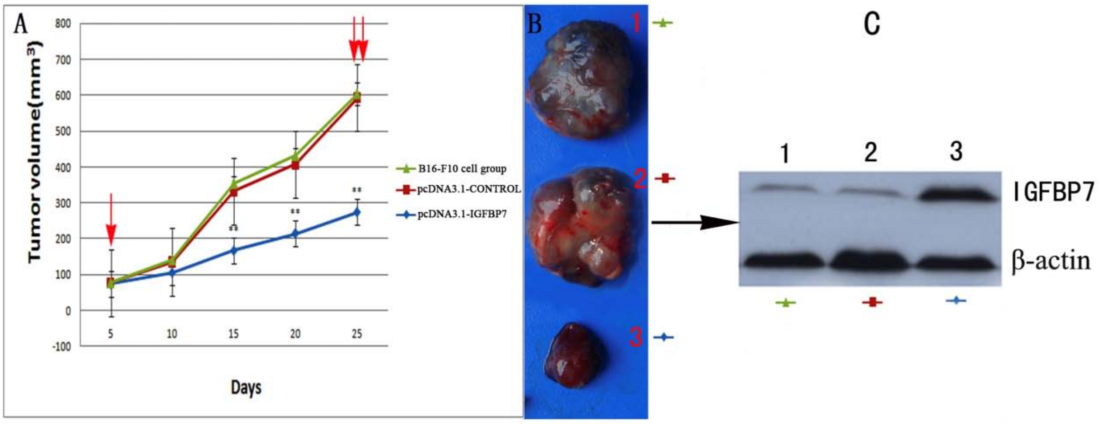

Figure S2 A Observations of the growth rate of MM xenograft after intratumoral injection with pcDNA3.1-IGFBP7 in vivo. Each point represents the mean volume ± SD of the tumor xenograft. Single red arrow represents the day of the Invivofectamine™ reagent–plasmid duplex complexes injection, and double red arrows indicate the time during which the mice were killed (**P < 0.01). B shows that at the time point of killing, the volumes of tumors in B16-F10, pc- CONTROL, and pcDNA3.1-IGFBP7group, are 587± 35 mm^3, 566 ±34 mm^3, and 256± 25 mm^3, respectively. C shows IGFBP7 expression determined by western blotting, 1 represents IGFBP7 expression in B16-F10 cell group, 2 represents, pcDNA3.1-CONTROL group, 3 represents pcDNA3.1-IGFBP7 group. Results show a typical experiment from three independent experiments.

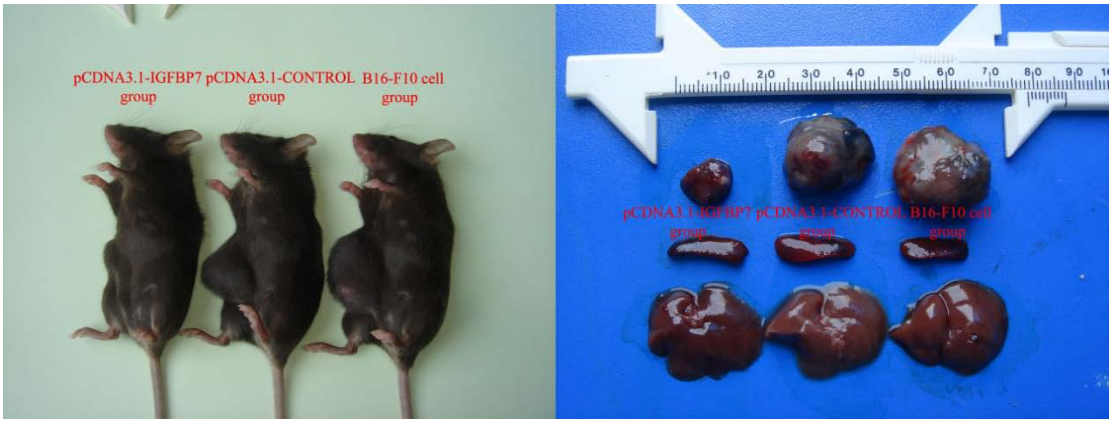

Figure S3 We have other two figures to reflect the volumes of tumors .

We were unable to provide the pictures of all the tumor tissues of each group together, because there were 12 mice in total in each group. In order to ensure the RNA, proteins within tumor tissue not to be degradated by RNAase or protease, and to maintain the structure of tumor tissue, the melanoma tissue had to be removed immediately and placed and fixed in 10% buffered neutral formalin. It took much time to kill all the mice by surgery and obtain melanoma specimens. Time was too tight for us to have all tumors photographed, so we have only one tumor tissue per group in the photo as a representative to reflect the volumes of tumors.
